# Stiffness Effects in Rocker-Soled Shoes: Biomechanical Implications

**DOI:** 10.1371/journal.pone.0169151

**Published:** 2017-01-03

**Authors:** Shih-Yun Lin, Pei-Fang Su, Chia-Hua Chung, Chi-Chun Hsia, Chih-Han Chang

**Affiliations:** 1 Department of Biomedical Engineering, National Cheng Kung University, Tainan, Taiwan; 2 Information and Communications Research Laboratories, Industrial Technology Research Institute, Tainan, Taiwan; 3 Department of Statistics, National Cheng Kung University, Tainan, Taiwan; Northwestern University, UNITED STATES

## Abstract

Rocker-soled shoes provide a way to reduce the possible concentration of stress, as well as change movement patterns, during gait. This study attempts to examine how plantar force and spatio-temporal variables are affected by two rocker designs, one with softer and one with denser sole materials, by comparing them with the barefoot condition and with flat-soled shoes. Eleven subjects’ gait parameters during walking and jogging were recorded. Our results showed that compared with barefoot walking, plantar forces were higher for flat shoes while lower for both types of rocker shoes, the softer-material rocker being the lowest. The plantar force of flat shoes is greater than the vertical ground reaction force, while that of both rocker shoes is much less, 13.87–30.55% body weight. However, as locomotion speed increased to jogging, for all shoe types, except at the second peak plantar force of the denser sole material rocker shoes, plantar forces were greater than for bare feet. More interestingly, because the transmission of force was faster while jogging, greater plantar force was seen in the rocker-soled shoes with softer material than with denser material; results for higher-speed shock absorption in rocker-soled shoes with softer material were thus not as good. In general, the rolling phenomena along the bottom surface of the rocker shoes, as well as an increase in the duration of simultaneous curve rolling and ankle rotation, could contribute to the reduction of plantar force for both rocker designs. The possible mechanism is the conversion of vertical kinetic energy into rotational kinetic energy. To conclude, since plantar force is related to foot-ground interface and deceleration methods, rocker-design shoes could achieve desired plantar force reduction through certain rolling phenomena, shoe-sole stiffness levels, and locomotion speeds.

## Introduction

In order to treat, rehabilitate, or carry out preventative care, clinicians often develop customized foot orthoses, the rocker-soled shoes, with a specially curved outer sole. These allow smooth progression through the stand phase of gait, reduce overbending of foot joints, and reduce local impacts and stresses on the foot [1]. The biomechanical effects of rocker-soled shoes are those of reinstating the lost motion of the lower extremities and reducing plantar pressure on specific parts of the foot [1]. Furthermore, for jogging, rocker-soled shoes changed dorsiflexion angles during heel-strike and mid-stance, reduced ankle plantar moments and first medial force peak [2]. Changes in plantar force distribution and ankle joint movement were found at rocker-soled shoes to be the reason for increases in variability at the microscopic level [3]. Therefore, rocker-soled shoes have thus become the most common type of specialized footwear [1, 4–8]. Moreover, to improve the gait of regular users, and reduce the chance of injury, numerous mass-producers of shoes also have designed soles with a slight curvature [3, 9].

The general structure of the rocker shoes is an outer surface curved in the front, and a flat middle section. The curve in rear section is especially intended to decrease pressure on heel strike, and reduce the need for ankle motion. This kind of heel rocker often focuses on the user who with special needs to alter their ankle kinetics [1, 7]. During normal roll-off, in a normal step, the progression of the line of gravity is slowest at the metatarsal heads, resulting in relatively long acting ground reaction forces during this rotation [10]. Therefore, the apex of the rocker shoes should be placed proximal to the area in which pressure relief is desired [1]. Furthermore, to achieve an ideal equilibrium, Chapman *et al*. [4] advised that an outsole design with a 95° apex angle, apex position at 60% of shoe length and 20° rocker angle could optimal offloading different regions of the forefoot.

Some studies evaluated the effect of the prolonged wearing of rocker-soled shoes [3, 11]. They compared the variability of biomechanical variables using rocker-soled shoes and conventional shoes. The results showed that prolonged wearing of rocker-soled shoes could increase postural control performance, decrease centre of pressure displacement, and decrease postural control system error. It is possible to carry out proprioception interference training using rocker-soled shoes, without taking specific device under certain circumstances. Other studies confirmed the hypothesis that the capacity of the plantar sole of the foot to convey somatosensory feedback about foot could be restored by inserting textured or noise-based insoles or changing footwear conditions [12–14].

From a biomechanical point of view, the goals in designing rocker-soled shoes include: (1) the curved surface at the front of the sole primarily functions to reduce stress and shear force in the push-off phase, reduce excessive bending of the metatarsal-phalangeal joint, and increasing forward thrust [1, 4, 8, 15]; (2) the curved surface at the rear of the sole primarily functions to reduce impact force during heel strike, and protects the heel bones and ligaments [1, 2]; (3) the flat middle part primarily supplies stability and support in midstance, as well as level plantar force [1, 2, 4, 6, 7].

In order to achieve optimal treatment results, clinicians consider not only the shape of curvature, but also the stiffness of sole to suit an individual patient’s feet. Furthermore, previous studies have indicated that in order to provide maximum surface area of contact with the sole of the foot, absorb shock, and provide optimal support, compound materials are recommended in shoe-sole design [1, 5, 16, 17]. For example, soft, moldable polyethylene has been recommended for contact with the sole of the foot. Firm molded cork or dense ethylene vinyl acetate (EVA) also has been recommended for the middle layer, to absorb reaction forces and provide excellent support. The lowest layer often utilizes fine, dense sponge rubber with anti-compression qualities, in order to absorb excessive shocks and provide good traction.

However, with so many studies having examined the differences between rocker shoes and traditional shoes, one major factor, the stiffness, which is changed by some specific portions seldom been discussed [6, 7]. Furthermore, to ensure the rolling-over effect, rocker shoes are usually designed by adding a stiffened rocker profile; few studies have quantitatively analyzed the biomechanical responses of different stiffness levels of rocker shoes.

In this study, we hypothesized that differing stiffness of rocker shoes will affect the stress concentration effect during locomotion. Moreover, the influences of different curvatures and locomotion speeds were also taken into consideration. Two rocker shoes were designed using softer and denser materials, respectively, and examined by comparing them with barefoot and flat-soled shoes. The specific aims were to test how and by what kinds of mechanisms rocker shoes with difference stiffness would influence the biomechanical parameters of gait pattern.

## Materials and Methods

### A. Participants

This study publicly recruited 11 male volunteers aged 30 to 40 (mean ± standard deviation values of demographic information: age 32.8±3.1 years, height 173.0±5.3 cm, body weight (BW) 72.1±6.9 kg and body mass index (BMI) 20.8±2.0 kg/m^2^). The recruitment criteria were: (1) the habit of wearing shoes since childhood, (2) absence of musculoskeletal disorders, and (3) BMI less than 27 kg/m^2^, since people whose BMI>27 kg/m^2^ are defined as obese by Taiwan official institutions [18]. Before testing, all volunteers were notified of potential risks and signed an informed consent document approved by the Institutional Review Board of Industrial Technology Research Institute (Case No. A003003).

### B. Equipment and instruments

#### 1) Shoes

In order to investigate how shoe sole design and materials influence gait pattern and plantar force, this study examined four foot-ground interface conditions: barefoot (Bare), flat shoes (Flat), rocker shoe-soles with a softer material in the midsole (Rocker-1), and rocker shoe-soles with a denser material in the midsole (Rocker-2). Furthermore, to provide appropriate size for all the subjects, three different shoe sizes were prepared (a total of 9 pairs of shoes).

**Fig 1:**
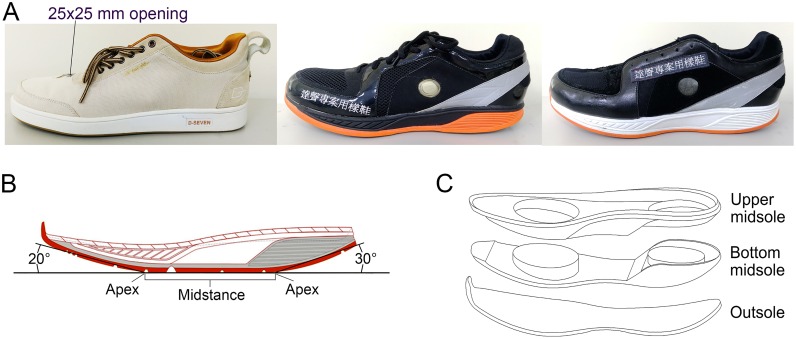
Three Experimental Shoe Types. (A) From left to right: Flat, Rocker-1, and Rocker-2; (B) Design of rocker shoe-sole; (C) Exploded diagram of rocker shoe-sole.

***Flat***: Soles have a flat design (D7-original, Da Sheng Corp., TW, the leftmost shoes in Fig 1A). Rubber is the only material used. Sole thickness at both heel and middle section is a uniform 25mm; front is 15 mm.

***Rocker-1***: All rocker shoes were designed and built from scratch; soles have a curved design (the middle shoes in Fig 1A). Outer surface of soles is curved upward at front and rear. Middle section is flat. Frontal rocker angle is upturn of 20°, rear rocker angle 30° of upturn [1, 4]. Thickness of soles at both heel and front of shoes is 15 mm; middle section is 30 mm. Apex dividing the front upturned section and middle section is designed to follow the line running from the 1st to the 5th metatarsal heads, which is about 60% of the shoe length from the heel and with about 95° apex angle (Fig 1B) [4, 19, 20]. Apex between rear upturned section and middle section is designed to fall under the junction of the plantar fascia and calcaneus, which is about 25% of the shoe length from the heel (Fig 1B) [1, 19–21]. The rocker radius is extended toward the front and back direction from the apex with a continuous curve [22]. Shoe soles have three layers using three different typically non-linear materials. All conform to ASTM (American Society for Testing and Materials) D638 and D3575 standards [23, 24], with material testing speeds of 12.5 mm/min. Layers are as follows: (A) Upper midsole: material is EVA I (young’s modulus: 0.77 MPa), a finer, denser material with good shock absorption characteristics. EVA I is used under the arch and at the upper sides of the sole. (B) Bottom midsole: material is EVA II (young’s modulus: 0.54 MPa), a softer material which allows for more change in shape, has a larger surface area for contact, and provides excellent support. EVA II is used under the metatarsal head of the forefoot, the calcaneus at rear of foot, and middle layer of the shoe-sole [17]. (C) Outsole: material is rubber (young’s modulus: 4.46 MPa), a denser material which provides good support and traction. The outsole is used for contact with the ground (Fig 1C) [25, 26].

***Rocker-2***: Design of the curved outer surface and overall structure of the sole is the same as the Rocker-1 (the rightmost shoes in Fig 1A). Two materials are used: (A) Upper midsole: material is EVA I; (B) Bottom midsole: material is also EVA I, the denser material than EVA II; (C) Outsole: material is rubber.

#### 2) Motion capture system

Kinematic data were acquired using 8 Vicon MX F20 (Vicon Motion Systems Ltd., Oxford, UK) infrared cameras which were rectified using static and dynamic calibration. Subsequently, motions were captured on infrared-retroreflective markers (14 mm) which were placed on the subject’s body and shoes. According to the position of the markers in space, movement tracks were traced by using the three-dimensional orientation of each segment of the human body.

#### 3) Vertical ground reaction force measurement system

Two AMTI BP600600 (AMTI, USA) force plates were used, one for each foot, to measure the vertical ground reaction force (VGRF).

#### 4) Plantar force measurement system

Pedar insoles systems (Noval electronics Inc., Germany) were used to measure the force applied from the sole of the foot to the shoe. Furthermore, to provide appropriate size for all the subjects, three different insole sizes were prepared.

### C. Procedure

Before each subject joined this study, we checked the raw movement tracks which were captured from the Vicon system and confirmed that all subjects were walking and jogging with rear-foot strike. Moreover, the test speed is lower than running and may not facilitate the fore-foot strike pattern [27, 28]. Each subject was asked to perform eight different tasks; the order of the four shoes (Bare, Flat, Rocker-1, and Rocker-2) and the two activities (walking and jogging) was randomly assigned. All subjects provided with appropriately sized shoes, socks, and Pedar insole systems; shoes were put on and tied by the same experimenter. In order to avoid slippage, the Pedar insoles were restricted by the socks in both Barefoot and shod conditions. Before each test, the Pedar insole system was calibrated according to the manufacturer’s instructions to eliminate the deviations generated from the shoes and socks.

After giving 10 minutes to get accustomed to each kind of shoes, additional familiarization was permitted until the subjects felt comfortable with the shoes. We defined jogging as a form of locomotion at a gentle pace between 2.00 to 2.50 m/s; walking at a slow pace between 1.00 to 1.50 m/s. Therefore, walking and jogging speeds were based on the subject’s normal, comfortable levels for those activities [9]; an eight-meter walkway was provided to reach the subject’s standard walking and jogging speed. Prior to carrying out each task, subjects were allowed as many practice trials as necessary and moved their starting position along the walkway so that they could land dependably on the two force plates, one for each foot, without altering their stride pattern. A trial was accepted if the subject completely hit the two force plates with the preferred leg without targeting.

In order to calculate the speed, values of distance difference and time difference needed to be obtained. We checked raw movement tracks which were captured from the Vicon system to obtain the difference in distance. The distance difference is defined as the distance between left and right heel-counter markers when a subject’s left and right feet separately land on their respective force plates. The time difference is obtained by measuring the time between the strike of the right and left foot on the force plate. After acquiring all the differences, we calculated the speeds as a subject’s feet were landing on the force plates along the walkway. The mean ± standard deviation values of average speed of walking and jogging were 1.38±0.09 m/s and 2.25±0.18 m/s, respectively.

To avoid the carry-over effect, all tests were divided by a rest period of at least 15 minutes in which shoes were not worn. Moreover, tests were divided into two sessions within one week (or at least two sessions within one day) in order to avoid long experiment times affecting performance. Before each test, subjects were assigned to put on the shoes and insole system one additional time, and we recalibrated the insole system between intervals. The sequence of the eight tasks was randomly assigned to each subject. All subjects had to perform three acceptable trials for each task.

Reflective markers were affixed to 16 bony landmarks on the body: bilateral anterior and posterior superior iliac spine, thighs, knees, tibias, ankles, heels, and toes; a 25x25 mm opening was also fashioned in the vamp, to allow placement of reflective markers to be placed on the bony landmark of the toe at the second metatarsal head. Another marker was placed on the heel counter, which represents the bony landmark of the heel; the thickness of the heel counter was deducted from calculations. Prior to the experiment the infrared camera was used, at a 100 Hz sampling rate, to define movement tracks and establish the seven segmental models (1 pelvic, 2 thighs, 2 shanks, 2 feet). Subjects were given sufficient time to familiarize themselves with the barefoot or shoe-shod condition, as well as with the equipment and testing environment [9, 29, 30].

**Fig 2:**
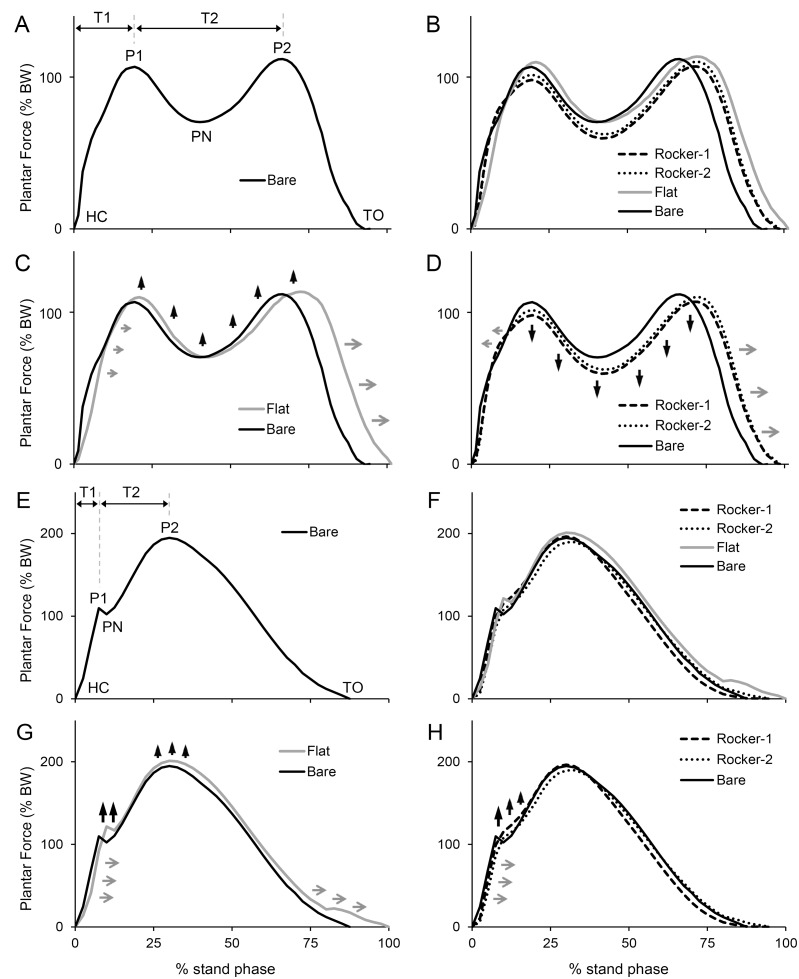
Normalized Force-time Curve. (A)-(D) and (E)-(H) show results for walking and jogging, respectively. (A) and (E) show Bare. Relevant events include: HC (heel contact), P1 (first peak), PN (peak negative), P2 (second peak), TO (toe off), T1 (HC to P1 duration), T2 (P1 to P2 duration). (B) and (F) show the four types of foot-ground interface. (C) and (G) provide a comparison of Bare and Flat. (D) and (H) compare Bare, Rocker-1, and Rocker-2. Changes in force are indicated by vertical black arrows; changes in time are indicated by horizontal gray arrows.

**Fig 3:**
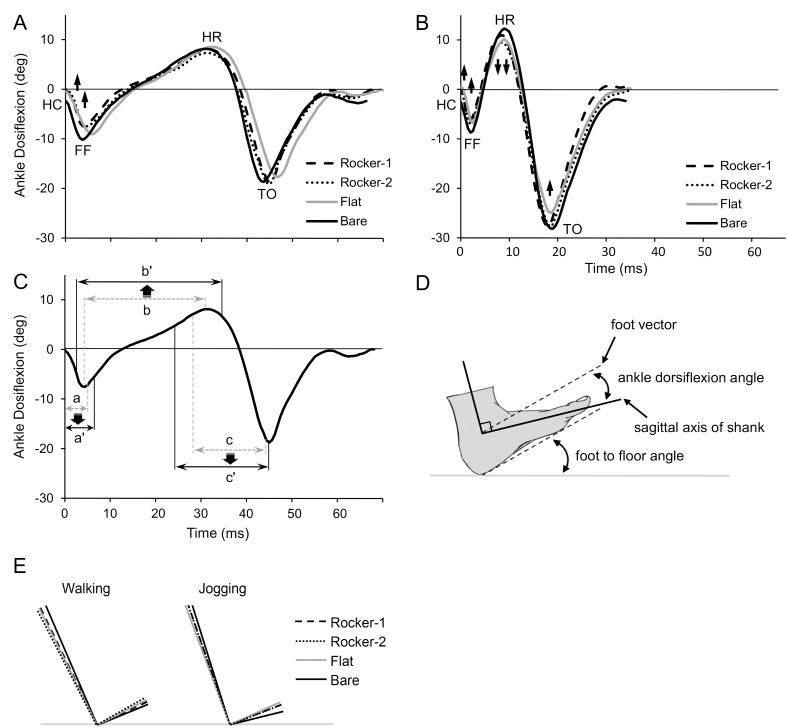
Changes in Angle Plotted Over Time. (A) and (B) show the ankle angle for walking and jogging, respectively; changes in angle are indicated by vertical black arrows. (C) shows the influence of rocker shoe-soles on gait in walking; a, b, and c indicate the heel, ankle, and forefoot rotation, respectively, produced by barefoot locomotion; a', b', and c' indicate the rear curve rolling, extended ankle rotation, and front curve rolling, respectively, produced by rocker shoes locomotion. Changes in length are indicated by vertical bold and black arrows. (D) and (E) indicate the ankle angle and foot to floor angle at HC.

To measure and subtract the difference between the Pedar insole system and force plate values, adjustments were made based on static calibration (measuring several fixed weights in a motionless state) as well as dynamic calibration (measuring barefoot locomotion) [31–33]. The adjustment equations were derived and the data of the Pedar insole system were calibrated test by test. According our calibration result, the linear relationships between the force and the value detected from insole pressure system are excellent; the R^2^ of left and right insole are 0.998 and 0.996, respectively. The experiment collected in total 264 gait cycles, and for each relevant event analyzed the parameters of plantar force, duration of event, and angle. In the gait cycle the relevant events were: heel contact (HC), foot flat (FF), heel rise (HR), and toe off (TO) (Figs 2A, 2E, 3A and 3B). For plantar force the relevant events were: first peak (P1), peak negative (PN), and second peak (P2) (Fig 2A and 2E).

### D. Analyzing parameters of event forces, duration, and angles

**Plantar force (PF, % BW)** was defined as the plantar force measured by the Pedar insole system divided by BW, expressed as a percentage (plantar force/BW*100).

**Difference of plantar force (DPF, % BW)** was defined as the force differences between plantar force measured by the Pedar insole system and VGRF measured by the force plate divided by BW, expressed as a percentage (difference of force/BW*100). The Bare value was taken to be a baseline.

**HC to P1 duration (T1, ms)** was defined as the duration of HC to P1 (Fig 2A and 2E).

**P1 to P2 duration (T2, ms)** was defined as the duration of P1 to P2 (Fig 2A and 2E).

**Difference of stand phase (DSP, % stand phase)** was defined as the duration differences of different events measured by the force plate divided by the overall stance phase duration, expressed as a percentage (difference of time/overall duration*100).

**Overall duration (OD, ms)** was **t**otal stance phase duration.

**Ankle dorsiflexion angle (deg)** was the angle of inclination between the line of the foot vector and the line of sagittal axis of the shank at HC. The foot vector is projected into the foot sagittal plane. The line of the foot vector is defined as the straight line joining the heel and toe marks. The line of the sagittal axis of the shank is defined as the straight line joining the knee and ankle joint centers. (Fig 3D).

**Foot to floor angle (deg)** was the angle of inclination between the line of the foot vector and floor at HC (Fig 3D).

### E. Statistical analysis

Linear mixed effect models, using an unstructured variance-covariance matrix, were used to compare mean changes for repeated observations on PF, DPF, DSP and OD, incorporating different types of foot-ground interface as covariate. The fixed effect is the action in which we take our primary interest. The subject effects are random effects. In particular, type III Wald F tests using Kenward-Rogers approximation for degrees of freedom were used to determine the effect of the type of shoes and get parameter-specific p-values for each model.

In addition, for comparing the mean difference between Rocker-1 and Rocker-2, paired t-tests were used if the normality assumption held. The mean and 95% confidence interval of all the differences were presented. Otherwise, the nonparametric method Wilcoxon signed rank test was applied. Each test was compared with the control at the 0.05 level of significance. Statistical procedures were performed with software R version 3.2.1 for Windows. The data including Rocker-1 and Rocker-2 were also evaluated for normality using the Shapiro-Wilk normality test. Note that we didn’t adjust multiple comparisons. Instead, we reported all raw p-values, and noted the statistical significance level 0.025≤*p*<0.05, 0.01≤*p*<0.025, and *p*<0.01. A general Bonferroni correction can easily be used based on the total number of tests that have been run. In this study, based on the power analysis, a minimum of 11 subjects was necessary to provide a statistical power of 80% to detect a 30% change in the pilot estimate of the parameter of interest [34].

## Results

### A. Plantar force and duration

Table 1 shows the estimated results for normalized plantar force and duration under the conditions of walking and jogging. Fig 2 shows the normalized force-time curve.

**Table 1:** Normalized Values for Plantar Force and Duration (Mean ± Standard Error).

	PF (% BW)			DPF (% BW)			DSP (% stand phase)		OD (ms)
Gait event	P1	PN	P2	P1	PN	P2	T1	T2	
**Walking**									
Bare	107.44±2.83	96.92±2.25	130.56±3.27	-	-	-	26.55±0.38	51.32±0.38	65.21±0.19
Flat	115.46±3.67***	98.58±2.85***	131.01±3.76	1.28±0.52**	2.33±0.57***	2.68±0.31***	29.21±0.91***	49.25±0.91***	74.28±0.46***
Rocker-1	91.23±3.67***	65.26±2.85***	104.84±3.76***	-30.55±0.52***	-28.28±0.57***	-24.59±0.31***	24.11±0.91***	52.10±0.91	73.36±0.46***
Rocker-2	101.06±3.67***^†††^	70.33±2.85***^†††^	120.16±3.76***^†††^	-23.47±0.52***^†††^	-15.78±0.57***^†††^	-13.87±0.31***^†††^	23.36±0.91***^†††^	53.07±0.91***^†††^	68.29±0.46***^†††^
**Jogging**									
Bare	105.95±4.57	103.01±4.89	192.31±6.55	-	-	-	5.17±0.57	31.23±1.10	32.49±0.92
Flat	128.47±8.33***	126.00±8.39***	198.11±9.01**	14.60±1.95***	3.64±1.82*	3.07±2.09	12.57±1.03***	25.50±1.80***	35.05±1.35***
Rocker-1	122.50±8.37***	121.16±8.43***	194.36±9.03	-4.79±1.97**	-12.17±1.84***	-12.24±2.12***	11.22±1.04***	27.00±1.81***	31.99±1.35
Rocker-2	116.55±8.32***^†^	114.96±8.39***^†^	186.47±9.01**^†††^	-5.02±1.95**	-13.24±1.82***	-13.16±2.09***	10.58±1.03***^†^	27.24±1.80***	33.02±1.35^†††^

PF, plantar force (plantar force/BW×100); DPF, difference of plantar force (difference of force/BW×100); DSP, difference of stand phase (difference of time/overall duration×100); OD, overall duration.

*, **, *** Indicate a significant difference between shod condition and Bare. The statistical significance levels were set at 0.025≤*p*<0.05, 0.01≤*p*<0.025, and *p*<0.01, respectively. Significant differences were taken to be the respective differences between each of the Flat, Rocker-1, and Rocker-2 values and the Bare value.

^†, †††^ indicate a significant different between Rocker-1 and Rocker-2. The statistical significance levels were set at 0.025≤*p*<0.05 and *p*<0.01, respectively. Unit of BW and force is N. Unit of time and duration is ms.

For walking, the PF were significantly larger for Flat and significantly smaller for rocker shoes than for Bare (Fig 2B–2D). It increased most obviously for Flat at P1, and decreased most obviously for rocker shoes at PN. For Rocker-1, PF were 16.21 (91.23–107.44) to 31.66 (65.26–96.92) % BW less than for Bare and the plantar force were 24.59 to 30.55% BW less than the VGRF. Furthermore, the DSP at T1 was significantly longer for Flat and significantly shorter for rocker shoes than for Bare. The OD values for the three types of shoe were all significantly longer than for Bare.

For jogging, except for Rocker-2 at P2, for all three shoe types, the PF was significantly greater than for Bare, with an especially large difference seen for Flat (Fig 2F–2H). Further, the plantar force was significantly larger for Flat and significantly smaller for rocker shoes than the VGRF. The values of DSP at T1 were significantly longer for all three types of shoe than for Bare. For the full OD, only Flat was longer than Bare.

### B. Comparisons of Rocker-1 and Rocker-2

For walking, the PF for Rocker-1 was significantly smaller than for Rocker-2 (Table 1). Further, the level of DPF reduction for Rocker-1 was significantly greater than for Rocker-2. For both Rocker-1 and Rocker-2, the greatest reduction of DPF was seen at P1. However, for jogging, the PF for Rocker-1 was significantly greater than for Rocker-2; the level of DPF reduction for Rocker-1 and Rocker-2 was similar. As seen in Table 1 for both walking and jogging, the DSP for Rocker-1 at T1 was significantly longer than for Rocker-2.

Table 2 shows the estimated results for comparing the mean difference between Rocker-1 and Rocker-2. For walking, the PF were significantly smaller for Rocker-1 in P1, PN, and P2 than for Rocker-2. However, for jogging, the PF were significantly larger for Rocker-1 in P1, PN, and P2 than for Rocker-2. Furthermore, no significant differences were observed for speed between Rocker-1 and Rocker-2 including walking and jogging.

**Table 2:** Comparison of Kinetics, Kinematics and Speed between Rocker-1 and Rocker-2.

Variables	Rocker-1^a^ [95% CI]	Rocker-2^a^ [95% CI]	Difference^a^ [95% CI]	*p*
**Walking**				
PF (% BW)				
P1	105.32 [102.37; 108.26]	109.27 [106.37; 112.17]	-3.21 [-5.37; -1.05]	0.004***
PN	73.71 [71.33; 76.08]	76.35 [74.50; 78.21]	-2.30 [-4.52; -0.08]	0.043*
P2	114.16 [110.90; 117.42]	117.55 [114.57; 120.53]	-2.57 [-4.84; 0.30]	0.027*
DSP (% stand phase)				
T1	20.50 [19.78; 21.22]	20.20 [19.45; 20.96]	0.23 [-0.60; 1.06]	0.578
T2^b^	58.82 [58.07; 60.32]	60.16 [58.21; 61.77]	-0.47 [-1.74; 1.05]	0.495
Angle dorsiflexion (°)				
HC^b^	-0.74 [-1.81; 0.10]	0.31 [-1.16; 1.74]	-0.47 [-1.59; 0.75]	0.484
FF	-7.54 [-8.56; -6.52]	-7.60 [-8.37; -6.83]	-0.17 [-1.08; 0.74]	0.703
HR	8.36 [7.17; 9.55]	7.32 [6.38; 8.26]	1.11 [0.26; 1.97]	0.012**
TO	-18.74 [-20.12; -17.36]	-18.97 [-19.95; -17.98]	0.46 [-0.51; 1.43]	0.347
Foot to floor (°)				
HC^b^	26.52 [24.49; 28.19]	27.78 [26.05; 29.47]	-1.04 [-2.29; 0.19]	0.102
TO	-70.84 [-71.98; -69.71]	-71.71 [-72.80; -70.61]	0.95 [-0.31; 2.21]	0.138
Speed (m/s)	1.39 [1.36; 1.42]	1.37 [1.34; 1.41]	0.02 [-0.02; 0.06]	0.247
**Jogging**				
PF (% BW)				
P1	122.44 [115.10; 129.79]	116.32 [110.26; 122.39]	6.94 [0.24; 13.65]	0.043*
PN	118.15 [109.36; 128.27]	115.35 [108.06; 123.91]	7.09 [2.49; 13.03]	0.005***
P2	193.15 [186.46; 199.84]	185.56 [179.78; 191.33]	9.18 [4.87; 13.48]	<0.001***
DSP (% stand phase)				
T1	8.56 [7.78; 9.34]	8.98 [8.26; 9.69]	-0.33 [-1.24; 0.58]	0.471
T2^b^	27.27 [25.71; 28.13]	26.97 [25.00; 28.57]	-0.36 [-1.58; 0.92]	0.577
Angle dorsiflexion (°)				
HC	0.71 [-0.76; 2.19]	0.06 [-1.09; 1.21]	0.01 [-1.07; 1.09]	0.983
FF	-6.28 [-7.57; -4.98]	-7.17 [-8.26; -6.07]	0.58 [-0.51; 1.66]	0.290
HR^b^	11.04 [9.52; 12.29]	10.24 [7.45; 11.35]	0.70 [-0.16; 1.60]	0.097
TO	-27.58 [-29.23; -25.92]	-27.69 [-29.06; -26.31]	0.29 [-0.88; 1.45]	0.622
Foot to floor (°)				
HC	-24.88 [-37.55; -12.21]	-22.79 [-34.90; -10.69]	-0.06 [-1.48; 1.36]	0.934
TO^b^	-67.69 [-70.36; -65.68]	-69.72 [-73.20; -66.99]	1.51 [-1.05; 3.94]	0.220
Speed (m/s)	2.28 [2.21; 2.35]	2.23 [2.16; 2.30]	0.05 [-0.01; 0.11]	0.104

*, **, *** Indicate a significant difference between Rocker-1 and Rocker-2. The statistical significance levels were set at 0.025≤*p*<0.05, 0.01≤*p*<0.025, and *p*<0.01, respectively.

^a^ Values include mean [95% confidence interval].

^b^ Indicates non-normal distributions. Non-parametric tests (Wilcoxon signed rank test) were chosen.

### C. Foot angle and gait pattern

Table 3 shows that in walking and jogging, at HC for Bare, both ankle dorsiflexion and foot to floor angle are the smallest (as shows in Fig 3A, 3B and 3F). Further, the increasing trends of these angles are greater for jogging than for walking; the increase for jogging with Flat shoes was especially pronounced. Fig 3C shows three ways that curved shoe-soles affect gait pattern (walking shown). These include the phenomena of rear curve rolling (Fig 3Ca') and front curve rolling (Fig 3Cc'), which are, respectively, analogous to heel rotation (Fig 3Ca) and forefoot rotation (Fig 3Cc). Furthermore, ankle rotation (Fig 3Cb) is both induced earlier and maintained longer, becoming extended ankle rotation (Fig 3Cb'). These three effects increase the time of simultaneous curve rolling and ankle rotation.

**Table 3:** Ankle Dorsiflexion and Foot to Floor Angle (Mean ± Standard Error).

	Ankle Dorsiflexion (deg)				Foot to Floor (deg)	
Gait event	HC	FF	HR	TO	HC	TO
**Walking**						
Bare	-2.05±0.89	-10.86±0.86	8.38±1.06	-18.50±1.00	21.35±1.25	-69.25±0.72
Flat	-0.24±1.52***	-9.38±1.42***	10.08±1.67***	-17.04±1.77	26.21±2.09***	-72.42±1.52***
Rocker-1	-0.18±1.52***	-7.52±1.43***	8.37±1.67	-18.73±1.77	25.85±2.07***	-70.85±1.5*
Rocker-2	0.03±1.53***	-7.51±1.43***	7.30±1.68^††^	-19.12±1.78	27.34±2.07***^††^	-71.70±1.5***
**Jogging**						
Bare	-2.35±1.34	-8.49±1.23	12.12±1.16	-28.59±1.10	14.79±1.50	-69.11±1.07
Flat	1.11±2.05***	-6.53±1.92***	10.20±1.83***	-25.24±1.98***	21.67±2.22***	-69.53±1.99
Rocker-1	0.29±2.09***	-6.49±1.96***	10.29±1.86**	-27.57±2.03	19.92±2.23***	-69.11±2.01
Rocker-2	0.39±2.06***	-6.85±1.92**	9.69±1.83***	-27.82±1.98	20.01±2.22***	-69.26±1.99

*, **, *** Indicate a significant difference between shod condition and Bare. The statistical significance levels were set at 0.025≤*p*<0.05, 0.01≤*p*<0.025, and *p*<0.01, respectively. Significant differences were taken to be the respective differences between each of the Flat, Rocker-1, and Rocker-2 values and the Bare value.

^††^ indicate a significant different between Rocker-1 and Rocker-2. The statistical significance level was set at 0.01≤*p*<0.025. Unit of angle is degree.

## Discussion

In order to effectively protect the feet and reduce the possible stress concentration during gait, numerous shoes have designed soles with different curvatures and materials. This study attempts to examine of how plantar force and spatio-temporal variables are affected by two new rocker designs, with softer and denser soles materials, respectively, by comparing with barefoot and flat-soled shoes. To the best of our knowledge, this is one of the first studies to systematically investigate the amount of change arising from the differing stiffness of rocker-soled shoes.

### Compensatory phenomena induced from shoes

Comparing with plantar force under Bare, in walking, Flat could significantly increase this force while both rocker designs could significantly reduce it (Table 1 and Fig 2B–2D). In both walking and jogging, DPF shows the plantar force for Flat was greater than the VGRF (Table 1). However, results were the opposite for rocker shoes. An especially large difference seen for Rocker-1 was 30.55% BW smaller for plantar force than for VGRF, indicating that force through rocker shoes is significantly reduced [2–4, 9].

A previous study proposed that VGRF and stepping speed have a linear relationship [35]; the other found that when jogging speed is increased from 1.5 m/s to 2.5 m/s, the peak pressure of the heel region shows the significant increase of 33% [36]. Furthermore, some studies proposed that the VGRF reflects the average acceleration of the whole body, and is not specific to the lower extremity. More interestingly, the VGRF is a composite of both high frequency “heel impact of foot” components and low frequency “center of mass of other body segments” components [37–39]. We hypothesize that for this study’s subjects, to prevent tripping on objects on the ground, as locomotion speed increased the angle of compensatory ankle dorsiflexion also increased especially in HC to HR duration. Sobhani *et al*. also found that the max ankle dorsiflexion angle of rocker shoe was increase from 13.3° in walking to 26.5° in running [39]. It can be inferred from this that, apart from the thickness of shoe-soles, the locomotion speed of body’s center of mass can also be an important factor to induce compensatory posture changes and increase VGRF.

Examining Table 3, it can be seen that wearing Flat in jogging, because of the thick heels and rapid pace, will induce increased compensatory ankle dorsiflexion during heel contact (Fig 3B and 3D); because of this, greater force will concentrate in the heel [21, 28, 40, 41]. Furthermore, the DPF for Flat represented that plantar force were 3.07–14.60% BW greater than VGRF. For Flat in walking, although there is a similar compensatory ankle dorsiflexion, because of the slower pace, the influence is less significant (Fig 3A and 3D); thus the DPF for Flat at P1 only represented an increase of 1.28% BW for plantar force than for VGRF, the influence is smaller than for jogging [21, 42].

Conversely, in walking the rocker-designed shoes could obviously absorb more impact force than for Bare. Primarily because the apex structure of the rocker-type soles was designed to be located where large forces are often applied to the foot [10]. During HC, rear curve rolling (Fig 3Ca') was produced along the bottom surface of the shoe-heel; this principle is analogous to the heel rotation (Fig 3Ca) along the calcaneus when barefoot. Further, the DSP of T1 for rocker shoes was significantly shorter than for Bare, indicating that ankle rotation is induced earlier in rocker-type soles, and increasing the time of simultaneous rear curve rolling (Fig 3Ca') and extended ankle rotation (Fig 3Cb'). In this way the kinetic energy of vertical impact force can be converted to rotational kinetic energy, effectively absorbing the vertical impact force [20, 28, 42, 43].

Moreover, during midstance phase, body weight will reduce the angle of upward curvature at the front and rear of the shoe soles, causing the middle section of the shoe-soles to bulge, forming the shoe-soles to spindle shape. Because of the spindle shape is in line with the natural curvature of the arch of the foot, the surface area of contact between the sole of the foot and the insole is increased, producing the phenomenon of weight redistribution. In this study, during peak negative, Rocker-1 and Rocker-2 were 31.66 (65.26–96.92) and 26.59 (70.33–96.92) % of BW less than Bare, confirming that rocker-type soles can effectively disperse plantar force [9]. During heel rise, rocker soles produced front curve rolling (Fig 3Cc') along the front outer surface of the shoe-sole; this principle is analogous to the forefoot rotation (Fig 3Cc) along the metatarsal head in barefoot locomotion. Also, ankle rotation finishes later and is thereby prolonged; the length of simultaneous extended ankle rotation (Fig 3Cb') and front curve rolling (Fig 3Cc') is increased, which can evoke more rotational reaction and absorb more vertical impact force [20, 28, 42].

However, with its greater speed and impact forces of jogging, the percentages of T1 values that occupy total stance phase duration for Rocker-1 and Rocker-2 were both decreased. The result was that during P1 impact forces produced greater speeds, rolling time was shortened, and the efficacy of rocker-type shoes was reduced; during this interval only 4.79 and 5.02% BW of impact force was absorbed. On the other hand, the percentages of T2 values for Rocker-1 and Rocker-2 that occupy total stance phase duration were slightly increased. These slightly longer times allowed the rocker soles to produce a partial effect.

### Comparisons of shoe sole materials between Rocker-1 and Rocker-2

Examining the differences between Rocker-1 and Rocker-2 (Table 1), in walking the PF for Rocker-1 was significantly smaller than for Rocker-2; the DPF was bigger for Rocker-1 than for Rocker-2. In jogging, however, the PF for Rocker-1 was significantly bigger than for Rocker-2, demonstrating that the materials used in shoe soles significantly affect the force received by the foot-sole. For the Rocker-1, which had a softer material under the metatarsal head and calcaneus, for walking, with its slower speed and smaller impact force, results for shock absorption were better. However, for jogging, because the transmission of force was faster, greater plantar force was seen in the softer material; results for higher-speed shock absorption were thus not as good. According to the results for DSP, T1 for Rocker-1 in both walking and jogging was significantly longer than for Rocker-2, showing that the softer material clearly lengthens the time from heel contact to the appearance of local maximum plantar force.

### Comparisons of four types of foot-ground interface

Examining Table 3, for both walking and jogging, during HC shoe-shod ankle dorsiflexion and foot-to-floor angle both presented as significantly greater than for Bare (Table 3 and Fig 3E); moreover, the increasing trend of those angles was greater for jogging than for walking. We can conclude that this may be due to the thickness of the shoe-sole causing the heel to contact the ground sooner, with the ankle not fully prepared to enter the flat-foot stage [21, 30]. It may also be due to the habit of wearing thick-heeled shoes, causing compensatory ankle dorsiflexion, with the degree of influence of this phenomenon increasing in proportion to step speed [36].

The findings of this study support those of previous studies. Bobbert *et al*. [42] concluded that during jogging, prior to heel contact joggers would utilize the “certain geometry of the body” and the anticipatory contraction of muscles to “land effectively.” Wit *et al*. [21] found that “in barefoot running, placement of the foot is significantly more horizontal than in the shod condition,” with 14° of difference between the two, this phenomenon occurring 0.03s before touchdown. Bonacci *et al*. [41] indicated that knee and ankle mechanics were different when barefoot than when wearing shoes, namely that there is a less dorsiflexed ankle at initial contact, and a less flexed knee during midstance.

Among the three types of shoe soles, because its heel was thickest, Flat most often produced early heel contact with the ground. This phenomenon causes vertical impact force to concentrate at the point of HC. In walking, for rocker-type soles, because the heel is thinner and more rounded, the DSP for T1 is significantly shorter than for Bare, and the earlier induction and greater speed of ankle rotation allows rotational motion to absorb force produced by vertical impact. However, with its greater speed of jogging, for rocker-type soles DSP during T1 was significantly longer than for Bare, reducing the effective absorption of vertical impact force [30]. Combining the above findings, we can conclude that it’s not just shoe-sole design that influences the angle at which the foot-sole contacts the ground; locomotion speed is also an important factor. Also, due to the faster step rate in jogging, the advantages provided by the structure and design of rocker-type soles are somewhat diminished, their shock-reduction effects being less good for jogging than for walking.

### Comparisons with other rocker-soled shoes

Many previous studies have been designed to quantify the changes in gait kinematics and kinetics caused by the use of rocker-soled shoes. Rigid double rocker-soled shoes [6] were verified as successfully maintaining a functional level in redistributing midfoot pressures without exacerbating hindfoot and forefoot pressures. The phenomena of increasing dorsiflexion in HC to FF duration, and decreasing dorsiflexion in HR to TO duration at the ankle joint during walking were also demonstrated in our study. Moreover, Boyer *et al*. [2] compared the commercialized rocker-soled shoe (Masai Barefoot Technologies, MBT) with the flat-soled shoe and demonstrated the result that the magnitudes of the first medial GRF peak and the peak anterior (push-off) force were lower for running in the rocker-soled shoe than in the flat-soled shoe. These results were also found in our experiment. Furthermore, they illustrated that the reduction in ankle range of motion in the early stance phase in the rocker-soled shoe is similar to that found in ankle foot orthoses. This phenomenon is verified by our experimental result that the rear curve rolling of the rocker-soled shoe is analogous to the heel rotation along the calcaneus when barefoot and can compensate the abrupt ankle plantar flexion moment during HC.

Another important relationship is the link between the rocker-soled shoe and the magnitude of the foot plantar force and ankle plantar flexion moment during stand phase of gait. Sobhani *et al*. [39] pointed out that shoes with frontal rocker design could induce a significant reduction in ankle power generation and plantar flexion moment impulse in late stance during both slow running and walking. Plantar flexion moment peak and impulse were also reduced by 11% and 12% in running, respectively [44]. Our study also supported this finding as shows in Table 1. The plantar force of walking and jogging for Rocker-1 (26.17 and 3.75% BW less) and Rocker-2 (10.85 and 11.64% BW less) were markedly less than Flat during P2 (the late stand phase.)

However, most rocker-soled shoes were made by adding a stiffened rocker profile; few studies focus on the material properties—such as the stiffness—of the sole. To the best of our knowledge, this is one of the first studies to systematically investigate the amount of change arising from the differing stiffness of rocker-soled shoes. Our experimental results supported the hypothesis that apart from the profile and thickness, different shoe-sole stiffness and stepping speeds both as significant factors in inducing compensatory postures in the lower extremities.

### Limitations

New designed shoes with different structures and materials require different lengths of time to become acclimated to, a previous study suggested that subjects needed to take at least 30 steps to in order to produce a good “average” step, and 166 steps to fully get used to new shoes [29]. Although this study gave subjects ample time to get used to the shoes and equipment, it did not force subjects to follow a strict step-count; this could be a source of error. Also, this study allowed subjects to determine comfortable, habitual walking and jogging speeds for themselves, and thus there was no uniform speed. According to our experiment results, all the values of jogging speed are within the range we defined. The issue about controlling the speed between conditions is an important factor and outght to be discuss in the future work.

In addition, because we only prepared three different sizes of shoes (a total of 9 pairs of shoes) and Pedar insole systems (a total of 3 pairs of insoles); we tried but ultimately couldn’t offer appropriate equipment and instruments for all of the female subjects. Therefore, subjects were all of the same gender and had been in the habit of wearing shoes since childhood, and thus utilized the rear-foot strike, which could account for some deviations in results. The outcomes of our research might not apply to other groups.

## Conclusions

In the gait cycle, plantar force is related to foot-ground interface and the method of deceleration.

Biomechanical implications of rocker-soled shoes arise not only from the characteristic of the rocker’s profile, but also from the shoe sole material stiffness and the locomotion mechanics. A rocker-soled structure designed with an optimal apex and stiffness suited to step speed can induce a longer period in which both curve rolling and ankle rotation occur simultaneously. The possible mechanism should due to the conversion from vertical kinetic energy into rotational kinetic energy, which can effectively absorb the plantar force produced by gait.
