# Correction: Stiffness Effects in Rocker-Soled Shoes: Biomechanical Implications

**DOI:** 10.1371/journal.pone.0176467

**Published:** 2017-04-19

**Authors:** Shih-Yun Lin, Pei-Fang Su, Chia-Hua Chung, Chi-Chun Hsia, Chih-Han Chang

The following information is missing from the Funding section: This study was supported by the Ministry of Science and Technology (MOST, https://www.most.gov.tw), Taiwan, R.O.C., under grant No. MOST 104-2221-E-006-265-MY2.
